# Impaired sense of agency and associated confidence in psychosis

**DOI:** 10.1038/s41537-022-00212-4

**Published:** 2022-04-02

**Authors:** Amit Regev Krugwasser, Yonatan Stern, Nathan Faivre, Eiran Vadim Harel, Roy Salomon

**Affiliations:** 1 Gonda Brain Research Center, Bar-Ilan University, Ramat Gan, Israel; 2 Psychology Department, University of Haifa, Haifa, Israel; 3 Univ. Grenoble Alpes, Univ. Savoie Mont Blanc, CNRS, LPNC, Grenoble, France; 4 Beer Yaakov-Ness Ziona Mental Health Center, Beer Yaakov, Israel; 5 Sackler Faculty of Medicine, Tel Aviv University, Tel Aviv, Israel

**Keywords:** Human behaviour, Psychosis

## Abstract

The Sense of Agency (SoA), our sensation of control over our actions, is a fundamental mechanism for delineating the Self from the environment and others. SoA arises from implicit processing of sensorimotor signals as well as explicit higher-level judgments. Psychosis patients suffer from difficulties in the sense of control over their actions and accurate demarcation of the Self. Moreover, it is unclear if they have metacognitive insight into their aberrant abilities. In this pre-registered study, we examined SoA and its associated confidence judgments using an embodied virtual reality paradigm in psychosis patients and controls. Our results show that psychosis patients not only have a severely reduced ability for discriminating their actions but they also do not show proper metacognitive insight into this deficit. Furthermore, an exploratory analysis revealed that the SoA capacities allow for high levels of accuracy in clinical classification of psychosis. These results indicate that SoA and its metacognition are core aspects of the psychotic state and provide possible venues for understanding the underlying mechanisms of psychosis, that may be leveraged for novel clinical purposes.

## Introduction

Psychosis is a severe psychiatric condition which includes numerous symptoms in which the delineation of the Self is compromised. Psychosis patients often report sensations of loss of control over their thoughts or actions, which has led to the suggestion that deficits in the demarcation of the Self constitute a core aspect of psychosis and schizophrenia spectrum disorders^1–3^. A central process giving rise to the sense of Self is the Sense of Agency (SoA), the feeling of control over one’s actions. Research has highlighted the role of SoA in delineating one’s bodily and mental functions from the environment and conspecifics, allowing one’s experience as a distinct embodied agent in the world^4–6^. Contemporary theories suggest that SoA is based on pre-reflexive predictive sensorimotor processes^5,7,8^, as well as explicit processes that take into account contextual and conceptual factors^9,10^. Within this theoretical framework, actions are accompanied by efferent copies that generate predictions regarding the expected sensory outcomes of these actions. Incoming afferent sensory information is then compared to the predictions. If the two match, the action is ascribed to the Self and accompanied by a SoA. These predictive mechanisms allow one to suppress the consequences of one’s actions both at the perceptual^7,11,12^ and the neural level^13–15^. However, if a mismatch occurs the sensory outcomes are ascribed to an external origin and are passed up the hierarchy to explicit processes that explain them in light of beliefs, knowledge and other contextual factors. Thus the integration of efferent predictive models and afferent sensory signals shape SoA and play a key role in delineating the Self^16,17^.

Disturbances of SoA are a striking aspect of psychosis, common across schizophrenia spectrum disorders^18–23^. It has been suggested that aberrant hierarchical prediction mechanisms underlie psychosis symptoms^24–26^ and specifically abnormal SoA^19,27^. Accordingly, psychosis patients exhibit reduced sensory and neural attenuation for actions^28,29^, impaired ability to predict the outcomes of their actions^22,30,31^ and erroneous explicit judgments of agency^18,32^. Thus, abnormal sensorimotor predictive mechanisms may induce inaccurate experiences of agency, causing confusion between self and externally induced sensations. Indeed, recent research has demonstrated a causal relationship between predictive processes and demarcation of the Self. For example, inducing tactile sensorimotor conflicts caused auditory self-discrimination deficits in first episode psychosis patients^33^, and psychosis-like symptoms in healthy participants^34,35^.

However, most studies of SoA have employed non embodied paradigms in which action-outcomes contingencies are acquired during the experiment (e.g., press a button—hear a tone). While these paradigms have enriched our understanding of learned action-outcome mechanisms, they do not tap into the strong predictive capacities afforded by a lifelong experience of controlling our bodies^36^. Thus, embodied SoA may differ in regards to the strength of the priors of the predictive processes^27,37^, and better capture psychotic patients’ anomalous self-experiences^3^.

While deficits in SoA have been found across the schizophrenia spectrum, it is yet unclear whether patients are aware of this impairment. Metacognitive deficits, involving lack of insight into their condition, are commonly found in psychosis and are associated with poorer prognosis^38–40^. However, recent research has shown that metacognitive capacities for some simple perceptual tasks do not seem to be deficient in schizophrenia patients^41,42^. Awareness of control over our actions, is critical for meaningful interactions with the world. While there have been some suggestions that SoA itself is a metacognitive mechanism^43^, and some contradictory suggestions^44^, to date there has been no study of metacognitive abilities of embodied SoA in psychosis.

The current pre-registered study examined embodied SoA and metacognition in psychosis patients and healthy controls (HC). We employed a virtual hand (VH) paradigm previously used in healthy participants^6,36^ in which we manipulate the sensorimotor correspondence between the participants’ real hand movement and the displayed VH’s movement by inserting a temporal or spatial alteration. First, we hypothesized that patients’ embodied SoA, operationalized as their ability to detect sensorimotor conflicts, would be impaired for both temporal and spatial alterations. Second, we hypothesized that their metacognition of SoA, operationalized as the correspondence between accurate sensorimotor conflict detection and associated confidence ratings, would be diminished compared to HC. Finally, in an exploratory analysis we examined whether we could accurately classify psychosis and HC participants based on task performance using an automated classifier, thereby probing the task’s clinical utility. Pre-registration, data and code are available online, see Data Availability section.

## Results

### Impaired SoA in psychosis patients

**Fig. 1:**
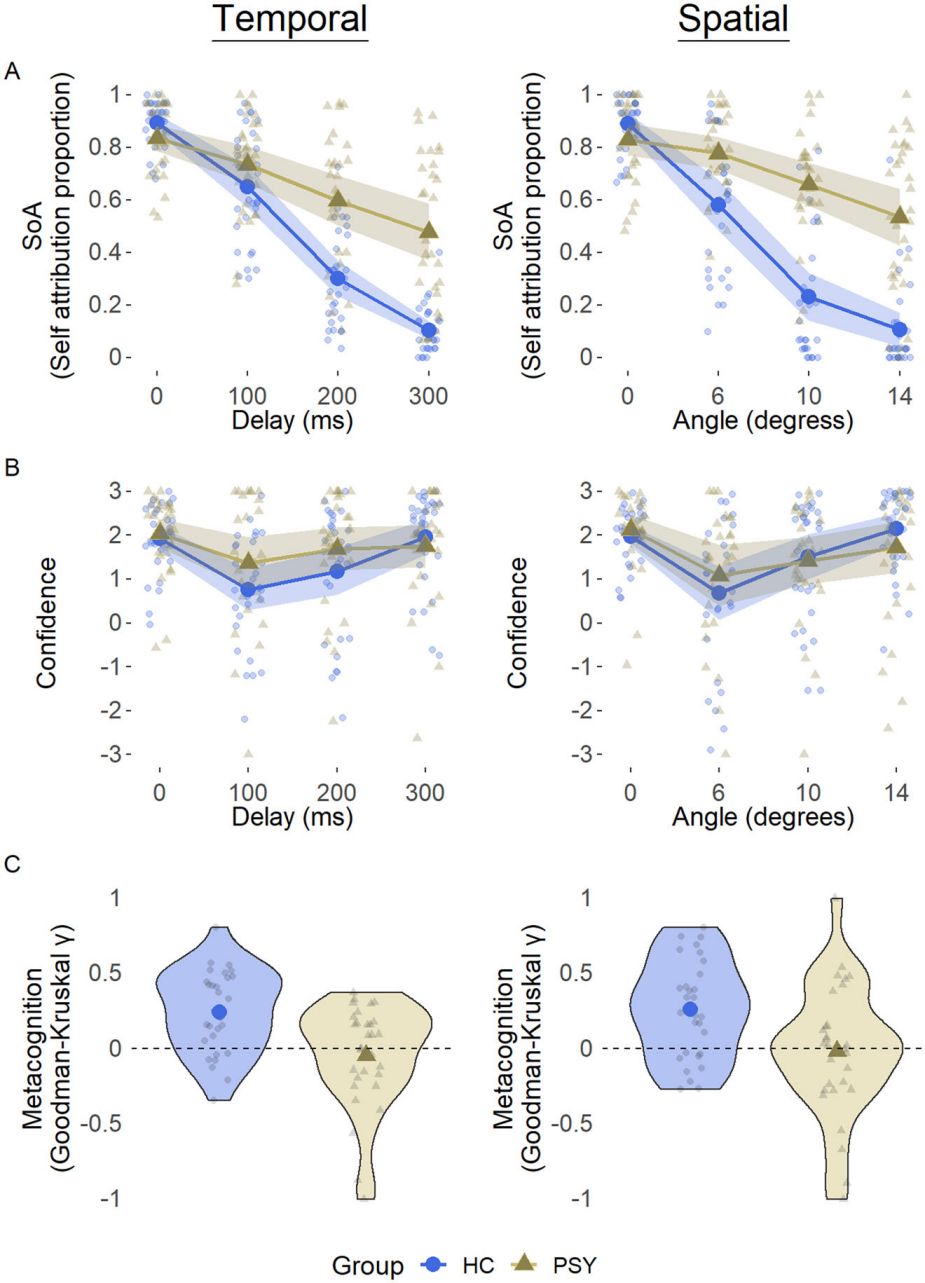
Group mean and individual ratings of SoA, confidence and metacognitive performance. **A** Self attribution in the temporal aspect (left) and in the spatial aspect (right). Shaded area represents 95% CI, large shapes represent group means. **B** Confidence in the temporal (left) and spatial (right) aspects following correct answers to the SoA question. **C** Distribution of metacognitive performance in the temporal (left) and spatial (right) aspects.

In line with our pre-registered hypothesis the psychosis group exhibited impaired SoA. The best model included the main effects of *Alteration Magnitude*, *Group* and their interaction. This model was better (Δ BIC = 3.9) than the next model that included the same terms in addition to *Aspect* and its interactions. The intercept and slope of *Alteration Magnitude* were included as random effects (see Supplementary material section A for full details of models). There was a significant main effect of *Alteration Magnitude* (β = −1.18, *p* < 0.0001, *Z* = 18.2, 95% CI [−1.31, −1.05]), such that as magnitude increased SoA ratings decreased across groups (see Fig. 1A). There was a significant main effect of *Group* (β = −0.61, *p* < 0.0001, *Z* = 5.9, 95% CI [−0.82, −0.41]), with the psychosis group showing an increased tendency to self-attribute the observed movements across the magnitudes of alteration. Notably, as predicted, there was a significant interaction between *Alteration Magnitude* and *Group* (β = −0.52, *p* < 0.0001, *Z* = 8.1, 95% CI [−0.65, −0.4]), resulting from the psychosis group’s moderate decrease in SoA ratings as alteration magnitude increased, in comparison to HC’s steep decrease in SoA as magnitude increased (see Fig. 1A). Importantly, similar results were obtained for different random effects structure (see Supplementary material section A). Complementing our finding of impaired SoA using mixed models, an independent samples Welch’s *t*-test of sensitivity and bias revealed that participants in the control group had higher sensitivity and lower bias than the psychosis patients (*d’*_Control_ = 1.8, *d’*_Psychosis_ = 0.76, *t*_56_ = 7.39, Cohen’s *d* = 1.9, *p* < 0.0001; *c*_Control_ = −0.43, *c*_Psychosis_ = −0.73, *t*_52_ = 2.88, Cohen’s *d* = 0.74, *p* < 0.01, see Supplementary Fig. S1). Furthermore, to address the heterogeneity of the psychosis group which might lead to a difference in SoA within this cohort, we compared SoA performance of a subgroup that contains schizophrenia, schizoaffective disorder and paranoid schizophrenia patients (*N* = 24) to SoA performance of a subgroup that contains brief psychotic disorder and bipolar disorder patients. This comparison did not yield any significant difference between these subgroups (all *p*-values > 0.14, see supplemental Fig. S2).

### Impaired metacognition in psychosis patients

In line with our pre-registered hypothesis metacognitive performance was impaired in psychosis patients. The best model included all main effects and interactions of *Alteration Magnitude*, *Group and SoA Accuracy* (i.e. was the SoA judgment correct), with very strong evidence (Δ BIC = 70) over a model that did not include *Group* and its interactions. The intercept and slope of *Alteration Magnitude* were included as random effects. Examining the winning model’s parameters, we found a significant three-way interaction between *Alteration Magnitude*, *Group* and *Accuracy* (β = 0.18, *p* < 0.001, *t* = 6.1, 95% CI [0.12, 0.24]). This interaction was driven by the psychosis group’s consistently higher confidence ratings despite their low levels of accuracy especially in trials with large alteration magnitudes (see Fig. 1B and Supplementary Fig. S3). Thus, the psychosis group exhibited impaired metacognitive capacities as their confidence ratings did not track their accuracy in comparison to the HCs. In addition, there was a main effect of *Alteration Magnitude* (β = −0.18, *p* < 0.001, *t* = 7.47, 95% CI [−0.13, −0.23]), reflecting the increased confidence when there was either an extreme alteration or none. Likewise, a main effect of *Accuracy* was found (β = −0.47, *p* < 0.001, *t* = 11.44, 95% CI [−0.55, −0.39]), reflecting that across groups, confidence was increased when SoA judgments were correct. In contrast, *Group* was not significant (β = −0.18, *p* = 0.07, *t* = 1.78, 95% CI [−0.39, 0.02]), thus overall confidence ratings between groups were not significantly different. Importantly, similar results were obtained for different random effects structure (see Supplementary material section A).

Further comparing the groups’ metacognitive performance using Goodman-Kruskal’s ranked correlation between confidence and accuracy, we found that the HC group exhibited a higher correlation (γ = 0.26, 95% CI [0.17, 0.35]) in comparison to the psychosis group (γ = −0.02, 95% CI [−0.12, 0.08]; see Fig. 1C), and this difference was significant (*t*_57_ = 4.27, *p* < 0.0001, Cohen’s *d* = 1.1). Examining whether each group’s ranked correlation significantly differed from zero (i.e. no correlation between accuracy and confidence) via a one-sample Student’s *t*-test, HC’s γ distribution was significantly higher than zero (*t*_*29*_ = 5.8, *p* < 0.001, Cohen’s *d* = 1.07), whereas the psychosis group’s was not significantly different from zero (*t*_*29*_ = 0.49, *p* = 0.63, Cohen’s *d* = 0.09). These findings complement the three-way interaction found in the mixed-models, demonstrating that psychosis patients exhibit impaired metacognition and their confidence ratings do not track their accuracy.

### Correlations between task performance and clinical measures

In psychosis patients, contrary to our hypothesis, we did not find a significant correlation between sensitivity and the total PANSS score (*r* = −0.03, *p* = 0.86) nor its subscales (see Table 1). Likewise, bias was not significantly correlated with the total PANSS score (*r* = 0.04, *p* = 0.84) nor its subscales. In an exploratory analysis, we found that metacognitive performance was significantly negatively correlated with the PANSS Positive subscale score (*r* = −0.47, *p* < 0.01, uncorrected for multiple comparisons), such that metacognitive performance was higher in patients with fewer positive symptoms.

**Table 1:** Correlations between clinical measures and sensitivity, bias & metacognition.

Group	Scale	d’	c	Goodman–Kruskal’s γ
Control	SPQ-B Cognitive-perceptual deficits	−0.32	0.24	0.21
	SPQ-B Interpersonal deficits	0.23	0.02	0.2
	SPQ-B Disorganization	−0.25	0.48**	0.11
	SPQ-B Total	−0.12	0.32	0.26
Psychosis	PANSS Positive	−0.16	0.01	−0.47**
	PANSS Negative	0.11	0.04	−0.17
	PANSS General	−0.08	0.05	−0.08
	PANSS Total	−0.03	0.04	−0.23

**p < *0.01* (uncorrected for multiple comparisons)

In healthy participants, contrary to our hypothesis, we did not find a significant correlation between sensitivity and schizotypy (i.e. total SPQ-B score) (*r* = −0.12, *p* = 0.53) nor its subscales (see Table 1). Likewise, bias was not significantly correlated with the total SPQ-B score (*r* = 0.32, *p* = 0.08), yet it was significantly correlated with SPQ-B Disorganization subscale (*r* = 0.48, *p* < 0.001, uncorrected for multiple comparisons).

### Group classifier

**Fig. 2:**
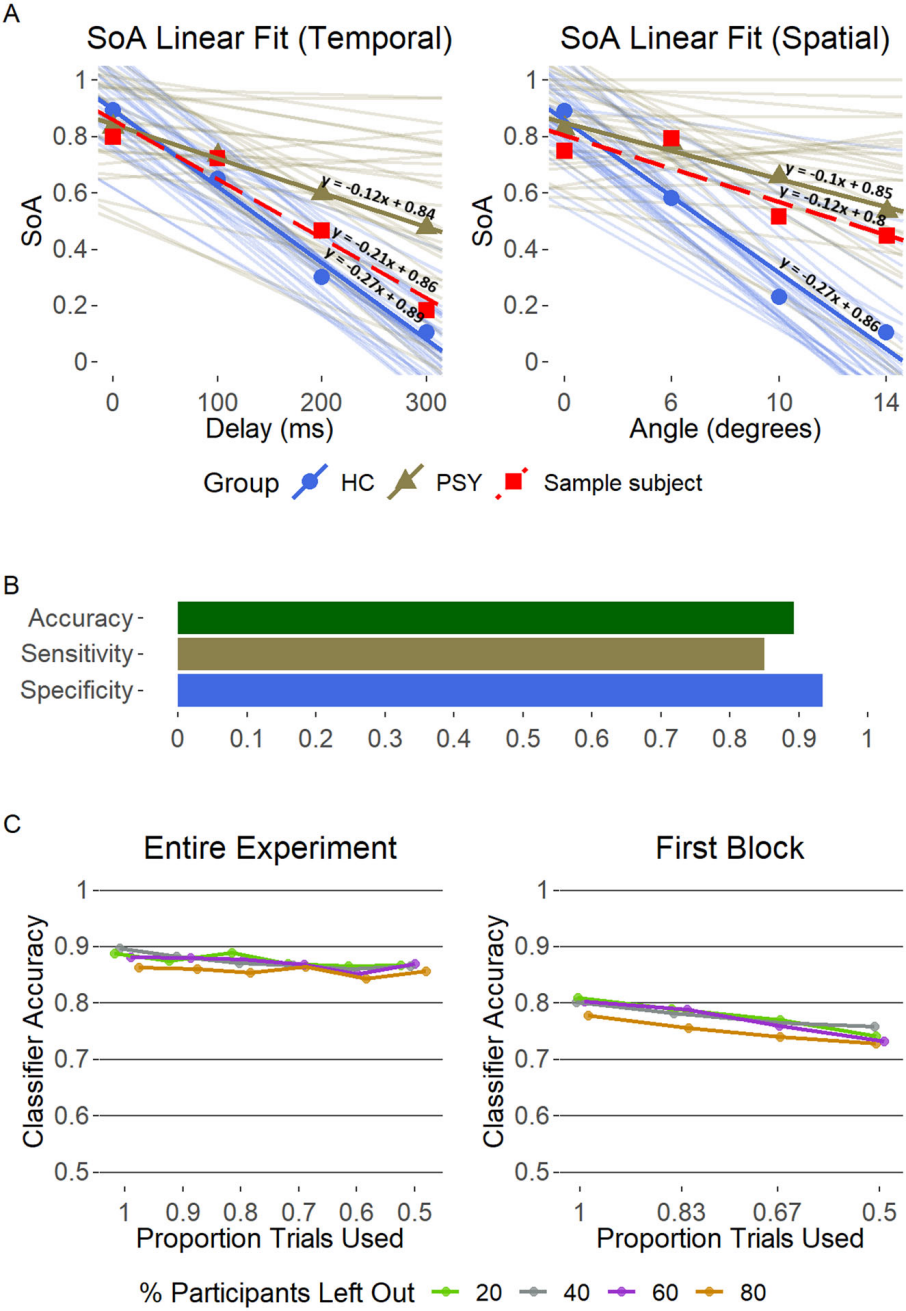
Group classifier performance. **A** Classification of two sample subjects (exemplar control participant and psychosis patient, linear fit in dashed red line), that are accurately classified as “control” (left) and “psychosis” (right). **B** Classifier performance, leaving out 20% of the participants (i.e. 6) in each iteration. Sensitivity is the percent of psychosis patients correctly classified, specificity is the percent of HC correctly classified. **C** Classifier accuracy across different proportions of participants left out and number of trials sampled. Trials were randomly sampled from the entire experiment (left), or from the first block (right).

Overall, the classifier was able to accurately classify participants in ~90% of the cases (see Fig. 2B). Using a two-sample Kolmogorov–Smirnov test for a difference between distributions, we found this accuracy rate to be significantly higher than chance level *(D* = 0.95, *p* < 0.001, tested by randomly labeling participants as control or patients, and comparing the accuracy rate of the classifier to the actual accuracy rate; See Supplementary Fig. S4). This finding was robust across different proportions of trials and participants left out, such that using only half the trials (i.e. 120 trials) and leaving out 80% of the participants (i.e. 24 out of 30 per group), only decreased the classifier performance to 85% accuracy (see Fig. 2C, left panel). To further examine the real-world applicability of our task, we also examined classification by sampling trials from the first block of the experiment only. This excludes the possibility that the high classification accuracy rates are dependent on the participants’ learning along the task. Using only the first 48 trials and leaving out 20% of the participants, we obtained 81% accuracy, that was reduced to 73% when using only 24 trials and leaving out 80% of the participants (see Fig. 2C, right panel).

## Discussion

Employing an ecological VR paradigm, we examined embodied SoA and associated confidence ratings in healthy and psychosis patients populations. Our results revealed several important findings. First, psychosis patients showed an extensive deficit in SoA, and were impaired in discriminating self from externally altered actions for both temporal and spatial alterations. Second, psychosis patients’ metacognition of SoA was impaired, and their confidence ratings did not track the accuracy of their SoA judgments. Finally, using a data driven approach to classify psychosis patients based on their embodied SoA task performance yielded high classification rates, suggesting that our task may be clinically useful in the detection and monitoring of psychotic states.

Psychosis patients showed a considerable deficit in their ability to judge whether the movement of the VH was identical to their actual movement or altered. For both temporal and spatial alterations their sensitivity to sensorimotor conflicts was significantly reduced compared to the control group, and they tended to erroneously attribute actions to themselves (i.e. an over-attribution of SoA). This impairment is in line with previous reports of reduced abilities to predict the outcomes of one’s actions in schizophrenia^22,30,31^. It has been previously suggested that abnormal temporal predictions and processing may underlie these SoA deficits^45–47^. However, the current findings indicate that embodied sensorimotor predictions in the spatial domain are also compromised^18,31^. In line with accounts highlighting disturbances of embodiment and the Self in psychosis^1,3^, our findings provide support for an impairment in the processing of the Self that extends across different perceptual dimensions.

In addition to SoA performance, we also investigated participants’ metacognition of SoA. While psychosis patients had comparable overall levels of confidence in their SoA judgments, this contrasted strongly with their low accuracy level. Converging evidence from the mixed model analysis and Goodman-Kruskal’s ranked correlations indicate that while the HC’s confidence tracked their SoA accuracy, this metacognition of SoA was absent in psychosis patients. Deficits of metacognitive capacities are well documented across the schizophrenia spectrum, as well as in individuals with genetic propensity for psychosis and schizophrenia due to 22q11.2 deletion syndrome^23^, and have been related to poorer outcomes in cognitive tasks, as well as lack of insight into one’s condition and deficits in emotion recognition^38,48–51^. However, recent work on perceptual metacognition indicates that when task difficulty is stringently controlled, metacognitive deficits in schizophrenia are small or even absent^41,42,52^. The current study examining embodied SoA in patients, found an extensive deficit in SoA discrimination, combined with high confidence in their judgments pointing to a considerable deficit in metacognition for SoA (although, one must take into account that first order performance was not equated here, which may account for some of the differences between the groups^42^). This suggests that in contrast to low-level perceptual metacognitive capacities which may be preserved, metacognition of SoA involving the integration of sensorimotor signals and higher-order constructs such as beliefs and intentions is severely impaired. This deficit is of clinical interest as the lack of SoA abilities compounded by their unawareness of this deficit, leading to high confidence, erroneous sensations of agency may relate to patients’ lack of insight into clinical symptoms such as hallucinations and delusions^38,53^. Interestingly, an exploratory analysis revealed a strong and significant correlation between Goodman-Kruskal’s γ metacognitive measure and positive symptoms in the psychosis patients group (*r* = −0.47, *p* < 0.01, see Table 1). Thus, of all experimental measures, metacognitive ability was most strongly related to psychosis symptoms, yet further research is needed to robustly examine this relation between metacognition of SoA and psychosis symptoms.

Psychosis patients showed a higher tendency to erroneously attribute actions to themselves. This self-attribution bias has been shown in previous studies with psychosis patients^18,21,54^ and in a human genetic model of psychosis^23^. It is intriguing in light of symptoms of psychosis such as passivity symptoms in which reduced agency is experienced^31^. It has been suggested that the over-attribution bias may originate from overweighting explicit top-down processes that take into account contextual information, intentions and beliefs in forming judgments of agency, thus compensating for the aberrant processing of sensorimotor signals in patients^19,28,29^. Indeed, previous work showed that reduced precision of sensorimotor predictive models may lead to overweighting top-down priors, causing an over-attribution of actions to the Self^24,27^. Our current finding of high subjective ratings of confidence despite low accuracy of SoA (i.e. impaired metacognition), supports this hypothesis that top-down explicit processes (i.e. ‘*I moved and saw a movement so it is likely me’*) may receive higher weighting despite impairments in sensorimotor prediction in psychosis.

The relation between clinical symptoms and SoA metrics revealed several interesting findings. Contrary to our pre-registered hypothesis, positive symptoms were not significantly correlated with SoA sensitivity (*r* = −0.16, *p* = 0.41, see Table 1) nor were SPQ-B perceptual deficits (*r* = −0.32, *p* = 0.08). An exploratory analysis revealed a correlation between the SPQ-B disorganization scale and bias (*r* = 0.48, *p* < 0.01). Indeed, previous studies have shown inconsistent correlations between prodromal symptoms^6,36^, psychosis symptoms^1,21,55^ and SoA measures. It should be noted that the current study’s sample size had low statistical power to detect such correlations.

Finally, we tested whether our embodied SoA paradigm might have clinical utility for identification and monitoring of psychosis. Using a classifier based on individual SoA performance, we were able to classify psychosis patients and controls with high levels of accuracy (~90%). Critically, this finding was robust when using only a small subset of trials or participants. This indicates that the difference in the tuning curve for the Self (i.e. the shape of the SoA slopes) is a strong predictor of psychosis across subjects, in line with accounts of an expanded sensorimotor temporal or spatial integration windows in psychosis, which may induce a wider “tuning curve” for the Self^17,20,22^. At the practical level, such computerized measurements could augment current in-person diagnosis of psychotic states by providing a telehealth option for online diagnosis and monitoring. Future studies may include a metacognition-based classifier (shown to be effective in separating schizophrenia patients and healthy controls in a social cognition paradigm^56^) in addition to SoA performance classifier to enhance classification accuracy even further. Employing multiple measurements could assess the relation of SoA and metacognition of SoA to patients’ clinical states over the course of hospitalization and recovery.

The current study suffers from several limitations. First, the psychosis cohort was not very large, only contained males and was diverse in its psychiatric diagnosis (see Table 2). However, we suggest that the robustness of our SoA findings, as well as the lack of differences between psychosis patients from the schizophrenia spectrum and those with psychosis diagnosed as part of a brief psychotic disorder or bipolar disorder, indicate that SoA and metacognitive deficits are a major feature of the psychotic state. Second, as we aimed to test SoA across different levels of sensorimotor ambiguity, our paradigm was not designed to stringently control for task difficulty and this limited our ability to employ novel metacognitive measures^35^. Future work on metacognition of SoA should control first order performance more stringently. Another limitation, the absence of a non-psychotic psychiatric control group, limits our ability to make claims regarding the specificity of SoA deficits to psychosis, as they putatively may be related to psychiatric illness in general. Future studies including psychiatric patients with a broad range of diagnoses are required to verify and validate the specificity of the relation between SoA abnormalities and psychosis. Finally, the control and psychosis groups were not matched for age, however no relations between age and any of the SoA or confidence measures were found (see Supplementary Table S6).

**Table 2:** Participants’ demographic and clinical characteristics.

Group	Diagnosis (N)	Age	PANSS Positive	PANSS Negative	PANSS Total
Psychosis	Schizophrenia (17)	30.6 (7.2)	14.6 (3.1)	16.6 (4.7)	64.3 (12.4)
	Schizoaffective disorder (5)	35 (13.5)	15.2 (2.6)	9.4 (2.4)	52.8 (7.5)
	Active psychosis (4)	29.3 (8.8)	17.3 (4.3)	16.5 (6.8)	67.5 (13.4)
	Paranoid schizophrenia (2)	28.5 (2.1)	12 (1.4)	16 (4.2)	59 (9.9)
	Bipolar disorder (2)	28 (8.5)	18 (0)	13.5 (6.3)	69.5 (19)
	*Mean*	*30.9 (8.3)*	*15.1 (3.2)*	*15.2 (5.2)*	*62.8 (12.4)*
Control	None (30)	*24.4 (3)*	–	–	–

Numbers represent the mean, numbers within the parenthesis represent the standard deviation.

*PANSS* = Positive and Negative Syndrome Scale.

In summary, employing an embodied virtual reality paradigm, we showed that psychosis patients are not only significantly impaired in their ability to discriminate their actions, but also show a substantial lack of awareness of this impairment. These results suggest deficits across multiple systems underlying SoA, including both low precision sensorimotor prediction mechanisms causing reduced sensitivity to deviations, as well as overreliance on top-down priors causing high confidence in erroneous judgments of agency. Importantly, patients’ insight to their difficulties in the demarcation of the Self may provide a foothold for understanding and treating Self disorders in psychosis.

## Methods

### Participants

#### Healthy controls

Thirty-four control participants that self-reported no psychiatric or neurological history from Bar-Ilan University participated in the experiment. Four participants were excluded from the analysis (see pre-registration and Supplementary material section C for criteria and details) leaving a total of 30 healthy participants (*mean age*: 24.4 years, *STD*: 3 years, *15 females*).

#### Psychosis patients

Thirty-one participants with psychosis from Beer Yaakov-Ness Ziona Mental Health Center participated in the experiment. One participant was excluded from the analysis (see pre-registration and Supplementary material section C for criteria and details) leaving a total of 30 psychosis participants (*mean age*: 30.9 years, *STD*: 8.3 years, all males. See Table 2 for clinical characteristics). Patients at the time of the experiment were hospitalized in a male-only department and under pharmacological treatment (see Supplementary Table S5 for medication details).

All participants gave written informed consent, were right-handed, with normal or corrected-to-normal vision and naïve to the purpose of the experiment. The experiment was performed in accordance with the ethical standards of the Declaration of Helsinki and the experimental protocols were approved by the Gonda Multidisciplinary Brain Research Center ethics committee (for HC) and by the Beer Yaakov-Ness Ziona Mental Health Center ethics committee (for psychosis patients).

### Experimental procedure

**Fig. 3:**
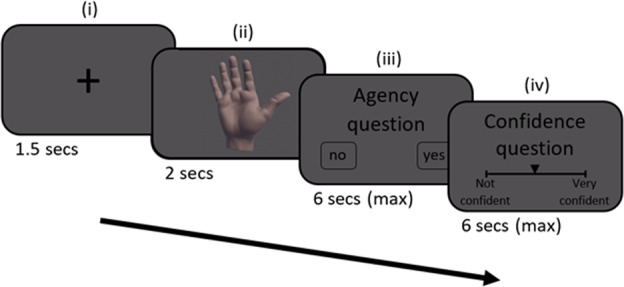
Trial flow. Each trial began with a fixation cross (i), followed by the VH presentation (ii), agency question (iii) and the confidence question (iv).

Participants’ right hand was occluded from their view and placed below a Leap Motion controller (Leap Motion Inc., San Francisco, CA) that tracked their hand’s movement. A realistic 3D VH that mimicked the real hand’s movement was displayed on a monitor (see Supplementary material section C and Krugwasser et al.^6^ for further details). Each trial began with a fixation cross, followed by a presentation of the VH during which participants performed a single bending movement with their index finger. In 25% of the trials, the VH’s movement was identical to the real hand’s movement, while in 75% of the trials a sensorimotor alteration was introduced. Three magnitudes of sensorimotor alterations were presented in temporal or spatial aspects. In the temporal aspect, the VH’s movement was delayed (100/200/300 ms^6,57,58^), and in the spatial aspect an angular deviation of the VH’s index finger’s was inserted (i.e. its lateral trajectory was diverged by 6/10/14° towards the thumb^6,18,59^). Importantly, only a single alteration (or none) was presented in each trial. Each magnitude of alteration per aspect was presented 30 times, in a random order across five blocks, resulting in a total of 240 trials. Following the VH presentation, participants responded to a Yes/No question “Was the movement of the VH identical to my movement?”, measuring SoA via the perceived congruence between the action and its outcome^6,18,36^. Participants then rated their confidence in the agency judgment on a continuous slider ranging from ‘Not confident’ (i.e. −3) to ‘Very confident’ (i.e. 3; see Fig. 3 for paradigm flow chart). Finally, the clinical symptoms of the psychosis patients were assessed using the Positive and Negative Syndrome Scale^60^ (i.e. PANSS), and HC participants completed the Schizotypal Personality Questionnaire–Brief Version^61^ (i.e. SPQ-B).

### Data analysis

Data was pre-processed using in-house Matlab scripts^62^. Following the pre-registration, trials in which no movement was made, camera malfunctioned, or participants failed to respond were removed from subsequent analyses (1.9% and 8.4% of the trials for HC and psychosis patients, respectively). Statistical analyses and visualization were performed in R^63^.

#### SoA

SoA (i.e. self-attributing the VH’s movement) was analyzed by comparing a series of logistic mixed-effects regressions implemented in the ‘*lme4*’ package^64^. Following Barr et al.^65^, we attempted to include maximal random effects that also allow for model convergence. Models were compared using the differences of their Bayesian Information Criteria^66^ (i.e. Δ BIC), with values between 2 and 6, between 6 and 10 and >10 considered as positive, strong and very strong evidence, respectively, in favor of the model with the lower value^67^. The winning model’s fixed parameters’ significance were derived using the Satterthwaite’s degrees of freedom approximation and type III error implemented in the ‘*lmerTest*’ package^68^. Signal detection measures of sensitivity and bias (*d’* and *c*, respectively) of SoA were also calculated, across magnitudes of alteration.

#### Confidence ratings

Similar to SoA, confidence was analyzed by comparing a series of linear mixed-effects regression models. Following an observed hyperbolic effect of *Alteration Magnitude* on *Confidence* (see Fig. 1B and Supplementary Fig. S3), a quadratic expansion of *Alteration Magnitude* was used as a fixed parameter. Metacognitive performance was assessed using Goodman-Kruskal’s gamma (γ) ranked correlations^69^ between confidence ratings and accuracy (calculated across the magnitudes of alteration for each participant). These correlations range from minus one to one, with a value of zero indicating that there is no association between accuracy and confidence.

#### Correlations with clinical measures

In line with our pre-registration, Pearson correlations were used to examine the relation between sensitivity and clinical ratings (i.e. PANSS scores for psychosis patients, and SPQ-B scores for HC). To further inspect the relation between performance and clinical measures, this analysis was supplemented with an exploratory analysis of the correlations between criterion, metacognitive performance and clinical ratings.

#### Clinical classification based on SoA performance

In an exploratory analysis, we examined the potential clinical utility of our SoA paradigm for classification of participants to psychosis or control groups based on their SoA performance. We developed an algorithm that classifies a given participant based on the comparison of his/her SoA judgments’ linear fit’s slope, to the mean slopes of both groups. Participants were classified to the group with the smaller Euclidean distance (from each group linear fit’s slope) combined across aspects of alteration (see Fig. 2A). The algorithm repeats this process 10,000 times, randomly leaving out the same proportion of participants from each group. Furthermore, we used the classifier with different proportions of left-out-participants as well as smaller subsets of trials for each participant (see Fig. 2C, left panel).

## Supplementary information

Revised supplementary material

Old supplementary material

Figure S1

Figure S2

Figure S3

Figure S4

## Data Availability

Pre-registration is available at https://bit.ly/2US57bX, data and code are available at https://github.com/amitrekru/SoA_Metacognition_Psychosis.
