# Supplementary material for: Impaired sense of agency and associated confidence in psychosis
Source: Schizophrenia (Heidelb). 2022 Apr 2;8(1):32. doi: 10.1038/s41537-022-00212-4 (PMC9261084; doi:10.1038/s41537-022-00212-4)
Supplement: Supplementary file 1 — Revised supplementary material [file 41537_2022_212_MOESM1_ESM.doc]

**Supplementary Material**

**Section A: Mixed Models**

**SoA**

| **Model's formula** | **BIC**  **RE = 1+Magnitude|ID** | **BIC**  **RE = 1+Aspect|ID** | **BIC**  **RE = 1|ID** |
| --- | --- | --- | --- |
| **SoA ~ Magnitude * Group + (RE)** | 13475 | 13205 | 13732 |
| **SoA ~ Magnitude * Aspect * Group + (RE)** | 13479 | 13230 | 13737 |
| **SoA ~ Magnitude * Group + Aspect + (RE)** | 13484 | 13214 | 13742 |
| **SoA ~ Magnitude + (RE)** | 13513 | 13735 | 14262 |
| **SoA ~ Magnitude + Group + Aspect + (RE)** | 13519 | 13722 | 14247 |
| **SoA ~ Magnitude + Aspect + (RE)** | 13522 | 13744 | 14271 |
| **SoA ~ Magnitude * Aspect + Group + (RE)** | 13525 | 13735 | 14277 |
| **SoA ~ Magnitude * Aspect + (RE)** | 13528 | 13754 | 14271 |
| **SoA ~ Group + (RE)** | 13578 | 17029 | 17398 |
| **SoA ~ Aspect + (RE)** | 13589 | 17060 | 17431 |

**Table S1.** Model comparison of ten SoA models using three combinations of random effects (RE). Left column is reported in the paper.

**Confidence**

| **Model's formula** | **BIC**  **RE = 1+Magnitude|ID** | **BIC**  **RE = 1+Accuracy|ID** | **BIC**  **RE = 1|ID** |
| --- | --- | --- | --- |
| **Confidence ~ Accuracy * Magnitude * Group + (RE)** | 48107 | 48034 | 48383 |
| **Confidence ~ Accuracy * Magnitude + (RE)** | 48177 | 48050 | 48468 |
| **Confidence ~ Accuracy * Magnitude + Group + (RE)** | 48185 | 48057 | 48476 |
| **Confidence ~ Group * Magnitude + (RE)** | 48329 | 48282 | 48642 |
| **Confidence ~ Aspect + Magnitude + (RE)** | 48381 | 48304 | 48692 |
| **Confidence ~ Magnitude + (RE)** | 48386 | 48311 | 48697 |
| **Confidence ~ Accuracy + Magnitude + (RE)** | 48390 | 48320 | 48706 |
| **Confidence ~ Group + Magnitude + (RE)** | 48394 | 48318 | 48705 |
| **Confidence ~ Accuracy + Group + Magnitude + (RE)** | 48399 | 48327 | 48714 |
| **Confidence ~ Aspect + (RE)** | 48496 | 48441 | 48804 |
| **Confidence ~ Accuracy + (RE)** | 48506 | 48453 | 48805 |
| **Confidence ~ Group + (RE)** | 48509 | 48453 | 48816 |

**Table S2.** Model comparison of twelve confidence mixed models using three combinations of random effects (RE). *Magnitude* fixed effect = a quadratic expansion of *Alteration Magnitude*, due to an observed hyperbolic effect of it on *Confidence* (see Fig 2B and 2S). Left column is reported in the paper.

Regarding metacognition, in addition to the effects described in the paper, there was also a significant two-way interaction between *Accuracy* and *Alteration Magnitude* (β = 0.45, *p* < 0.001, *t* = 15.1, 95% CI [0.39, 0.51]) reflecting the upward concave of *Alteration Magnitude* on correct trials as opposed to the downward concave on incorrect trials (see Fig. 1b and supplemental Fig. S2). Contrary to our expectation, the interaction between *Group* and *Accuracy* was not significant (β = 0.03, *p* = 0.5, *t* = 0.68, 95% CI [0.05, 0.11]).

**Section B: Data Analysis**

**Mixed Measures ANOVA**

To test for differences in SoA between the groups, we employed a 2×2×4 mixed measures ANOVA with *Aspect* and *Alteration Magnitude* as within-subject factors and *Group* as between-subject factor (when required Greenhouse-Geisser corrections were applied). Trials without an alteration (i.e., alteration magnitude 0) were randomly split into one of the two aspects. Null effects were assessed by a Bayesian mixed measures ANOVA and Bayesian paired *t*-test (a *BF_10_* above 3 indicates substantial support for the alternative and a *BF_10_* less than 0.33 indicates substantial support for the null^1^)

To test for differences in confidence between the groups, we employed a 2×2×4 mixed measures ANOVA with *Aspect* and *Alteration Magnitude* as within-subject factors and *Group* as between-subject factor (when required Greenhouse-Geisser corrections were applied).

**Results**

***ANOVA - SoA.*** The mixed measures ANOVA revealed a main effect of *Group* (*F*(1,58) = 42.08, *p* < 0.001, *η^2^* = 0.42) driven by overall higher SoA ratings in the patients group than in the control group (*M*_Control_ = 0.46, 95% CI [0.42, 0.5]; *M*_Psychosis_ = 0.68, 95% CI [0.65, 0.71]). We also found a main effect of *Alteration Magnitude* (*F*(3,174) = 302.9, *p* < 0.001, *η^2^* = 0.44) driven by a decrease of SoA ratings as alteration magnitude increased (*M*_alt0_ = 0.86, 95% CI [0.84, 0.88]; *M*_alt1_ = 0.69, 95% CI [0.65, 0.73]; *M*_alt2_ = 0.45, 95% CI [0.4, 0.5]; *M*_alt3_ = 0.31, 95% CI [0.25, 0.36]). Importantly, an interaction between *Alteration Magnitude* and *Group* was found (*F*(3,174) = 54.31, *p* < 0.001, *η^2^* = 0.078), indicating that SoA ratings in the psychosis group exhibited a more gradual decrease as alteration magnitude increased relative to the control group (see Table S3). SoA ratings between aspects were similar (*M*_Temporal_ = 0.57, 95% CI [0.54, 0.61]; *M*_Spatial_ = 0.58, 95% CI [0.54, 0.63]), and a Bayesian analysis further demonstrated strong evidence in favor of the null hypothesis that the aspects are similar (*BF_10_* = 0.11, *F*(1,58) = 0.25, *p* = 0.62, *η^2^* = 0.0001).

| **Group**  **Alt.**  **Magnitude** | **Control** | **Psychosis** |
| --- | --- | --- |
| **0** | 0.89 [0.87, 0.91] | 0.83 [0.8, 0.87] |
| **1** | 0.62 [0.56, 0.68] | 0.76 [0.71, 0.8] |
| **2** | 0.27 [0.21, 0.32] | 0.63 [0.57, 0.69] |
| **3** | 0.11 [0.07, 0.14] | 0.51 [0.43, 0.58] |

**Table S3.** Mean SoA of each group in each alteration magnitude [95% CI].

***Confidence.*** The mixed measures ANOVA for confidence ratings following a correct answer revealed a main effect of *Alteration Magnitude* (*F*(3,165) = 22.5, *p* < 0.001, *η^2^* = 0.09) driven by different levels of confidence between alteration magnitudes. No main effect of *Group* was found (*F*(1,55) = 0.42, *p* = 0.52, *η^2^* = 0.008), with the Bayesian analysis providing strong evidence for the lack of difference in overall confidence between groups (*BF_10_* = 0.017). The interaction between *Alteration Magnitude* and *Group* was significant (*F*(3,165) = 4.31, *p* < 0.01, *η^2^* = 0.017), demonstrating the change in confidence levels between alteration magnitudes was different for each group. There was no main effect of *Aspect* (*F*(1,55) = 0.21, *p* = 0.65, *η^2^* < 0.001, *BF_10_* = 0.13), nor was its interaction with *Group* significant (*F*(1,55) = 1.19, *p* = 0.28, *η^2^* = 0.001, *BF_10_* = 0.004), suggesting that confidence following a correct answer did not differ between aspects.

| **Group**  **Alt.**  **Magnitude** | **Control** | **Psychosis** |
| --- | --- | --- |
| **0** | 1.83 [1.56, 2.11] | 1.99 [1.66, 2.32] |
| **1** | 1.42 [1.11, 1.73] | 1.91 [1.59, 2.24] |
| **2** | 1.5 [1.16, 1.85] | 1.85 [1.54, 2.16] |
| **3** | 2.02 [1.73, 2.31] | 1.87 [1.51, 2.24] |

**Table S4.** Mean confidence of each group in each alteration magnitude [95% CI].

*****[Placeholder for figure S1]*****

**Figure S1.** SoA Sensitivity (*d'*) and bias (*c*) of both groups.

*****[Placeholder for figure S2]*****

**Figure S2.** SoA ratings of HC and two patients subgroups - schizophrenia spectrum and non schizophrenia spectrum patients

*****[Placeholder for figure S3]*****

**Figure S3.** Confidence for incorrect SoA judgments.

**Section C: Participants & Experimental Paradigm**

**Medications**

Psychosis patients were treated with one or more of the following medications:

Risperdal, Ariply, Depakote, Zyprexa, Abilify, Cymbalta, Clopixol, Haldol, Depalept, Seroquel, Entumin, Lithium, Modecate and Leponex.

| Participant # | Medications | |
| --- | --- | --- |
| **1** | T. Risperdal 3mg |  |
| **2** | T. Ariply 10mg | T. Depakote 1000mg |
| **3** | T. Abilify 15mg | T. Cymbalta 60mg |
| **4** | T. Zyprexa 20mg | Clopixol depot 200mg |
| **5** | T. Zyprexa 20mg |  |
| **6** | T. Haldol 5mg | Depalept 100mg |
| **7** | T. Seroquel 800mg |  |
| **8** | T. Zyprexa 20mg |  |
| **9** | T. Zyprexa 20mg | Clopixol depot 200mg |
| **10** | T. Zyprexa 20mg | Haldol dec 100mg |
| **11** | T. Risperdal 3mg |  |
| **12** | T. Risperdal 4mg | Entumin 40mg X2 |
| **13** | T. Risperdal 3mg |  |
| **14** | T. Haldol 10mg |  |
| **15** | T. Zyprexa 20mg | Depalept 750mg |
| **16** | T. Haldol 10mg |  |
| **17** | T. Zyprexa 20mg | Lithium 900mg |
| **18** | T. Zyprexa 20mg |  |
| **19** | Haldol dec 100mg |  |
| **20** | T. Zyprexa 20mg | Depalept chrono 1500mg |
| **21** | T. Risperdal 4mg |  |
| **22** | T. Zyprexa 20mg | Depalept 1250mg |
| **23** | T. Risperdal 4mg |  |
| **24** | Clopixol dept 200mg |  |
| **25** | T. Zyprexa 20mg | Clopixol dept 200mg |
| **26** | T. Zyprexa 20mg | Clopixol dept 200mg |
| **27** | T. Depalept 1200mg | Modecate 25mg |
| **28** | T. Clopixol 200mg |  |
| **29** | T. Zyprexa 20mg | T. Depalept 1000mg |
| **30** | T. Zyprexa 20mg | T. Leponex 240mg |

**Table S5.** Psychosis patients' medication information.

**Diagnosis**

Bipolar patients were diagnosed as suffering from a bipolar disorder including a current episode manic with psychotic feature.

**Exclusion Criteria**

The exclusion criteria for both groups were pre-registered.

Healthy participants:

Participants who failed to complete at least 80% of the trials (one participant) or participants for whom the Leap controller malfunctioned (as reported in the controller logs) in more than 10% of the trials (three participants).

Psychosis patients:

Participants who failed to complete at least 50% of the trials or participants that were unable to comply with the task requirements as evidenced by the anatomical alteration test (one participant, see Screening task for psychosis patients).

**Setup**

The experiment was administered via custom-made software (LeapV2, Unity 5.6.1, see Krugwasser et al.^2^ for a similar setup), running on a computer with an Intel core i7 processor and 16 GB of RAM. The experiment was displayed on a 24 inch (Dell P2417H) monitor, at a 1920x1080 resolution (refresh rate: 60Hz). 3D modelling and motion tracking of participants’ hand were obtained using a Leap Motion controller (Leap Motion Inc., San Francisco, CA), creating a realistic VR model of the participants’ hand and movement in real-time. Participants placed their right hand facing upwards, directly beneath the Leap Motion controller, while their hand was occluded from their vision via a barrier. They responded to task questions with their left hand via a numeric keyboard with three available keys – left, right and enter.

**Screening task for psychosis patients**

Psychosis participants also performed a brief screening task, with a pre-registered exclusion criterion. This task follows the same paradigm but with anatomical alteration (i.e., they moved their index finger and saw their middle finger move instead), rather than a temporal or spatial alteration. This paradigm corresponds to the anatomical condition used in experiment 1 in Krugwasser et al.^2^. The task consisted of 48 trials, with an anatomical alteration occurring in half of the trials. Patients that did not show a decrease of at least 20% in SoA between the unaltered condition and the anatomical alteration condition were excluded from the analysis.

**Correlation between age and performance**

To check for a possible effect of age on performance in the task, we correlated age with sensitivity, bias and metacognitive performance within each group. A significant correlation might suggest that performance is age-dependent to some extent. All correlations were low and non-significant, suggesting age did not affect performance.

| **Group**  **Measure** | **Control** | **Psychosis** |
| --- | --- | --- |
| **d'** | r = 0.15, p = 0.42 | r = -0.02, p = 0.93 |
| **c** | r = 0.16, p = 0.41 | r = 0.08, p = 0.68 |
| **GK** | r = 0.2, p = 0.29 | r = 0.23, p = 0.22 |

**Table S6.** Correlation between participants' age and sensitivity, bias and metacognitive performance.

**Section D: Classifier Description**

| **Actual**  **Classifier decision** | **Psychosis** | **Healthy** |
| --- | --- | --- |
| **Psychosis** | True Positive | False Positive |
| **Healthy** | False Negative | True Negative |

**Table S7.** Classifier's confusion matrix.

Classifier accuracy was calculated as:


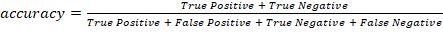
,

classifier sensitivity was calculated as:
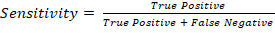
, classifier specificity was calculated as:
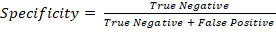


*****[Placeholder for figure S4]*****

**Figure S4.** Classifier accuracy distributions for the actual results and for the actual results with randomly scrambled labels. Dashed lines indicate the accuracy rates in both conditions.

**References**

1. Dienes, Z. Using Bayes to get the most out of non-significant results. *Front. Psychol.* **5**, (2014).

2. Krugwasser, A. R., Harel, E. V. & Salomon, R. The boundaries of the self: The sense of agency across different sensorimotor aspects. *Journal of Vision* **19**, 14–14 (2019).
