# Supplementary figures and images for: Impaired sense of agency and associated confidence in psychosis
Source: Schizophrenia (Heidelb). 2022 Apr 2;8(1):32. doi: 10.1038/s41537-022-00212-4 (PMC9261084; doi:10.1038/s41537-022-00212-4)

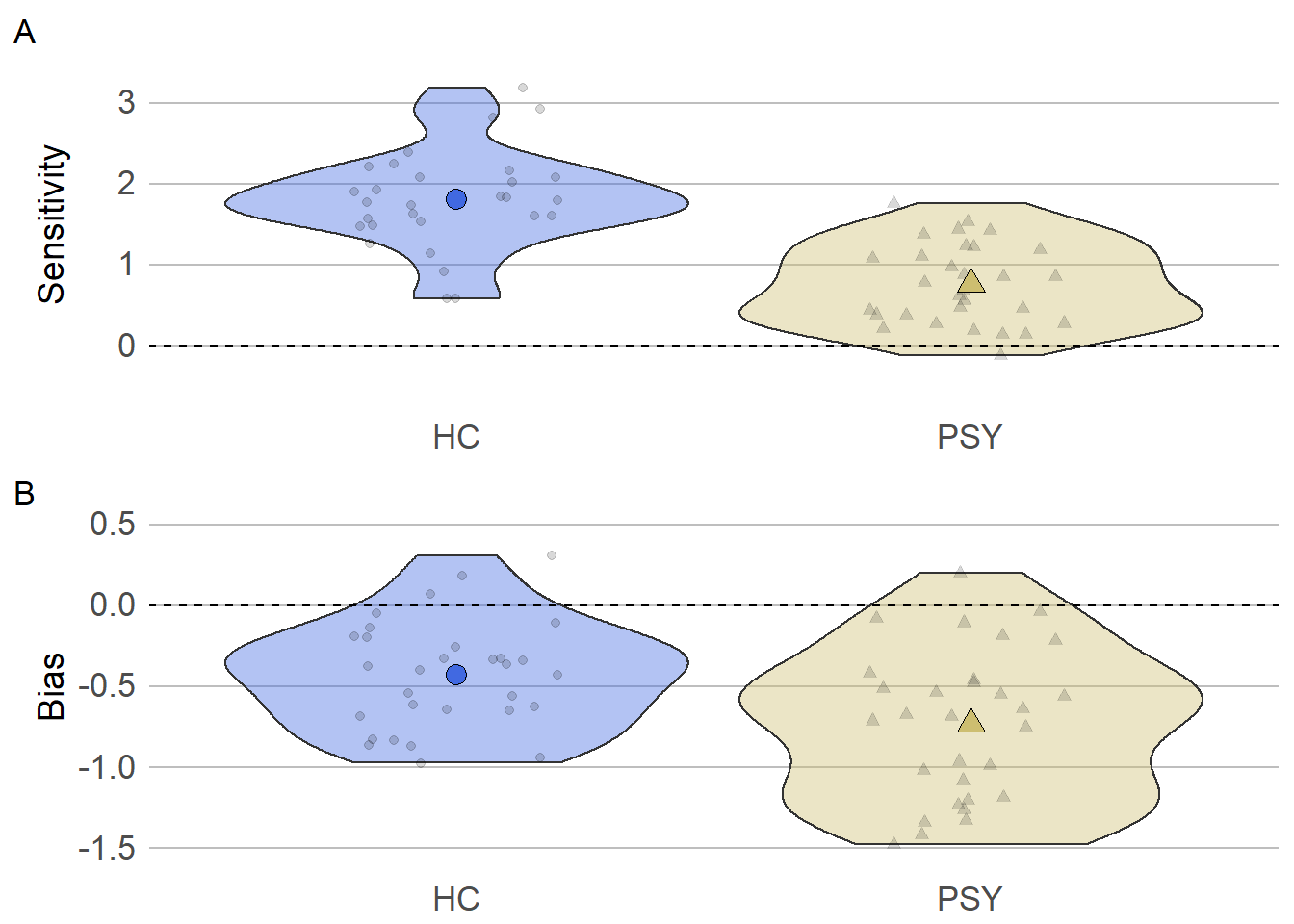

Supplement: Supplementary file 3 — Figure S1 [file 41537_2022_212_MOESM3_ESM.tif]

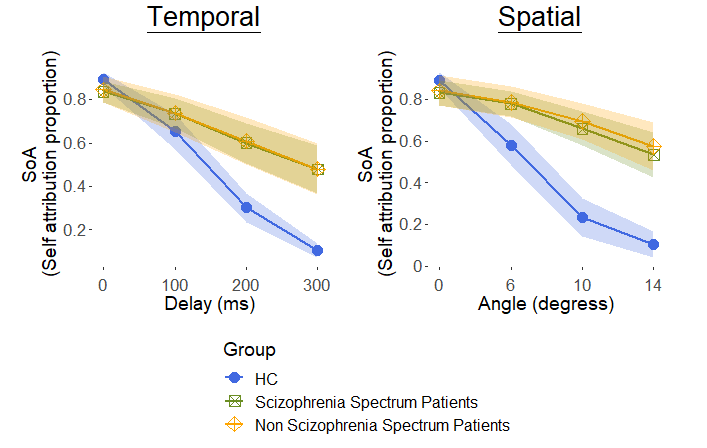

Supplement: Supplementary file 4 — Figure S2 [file 41537_2022_212_MOESM4_ESM.tif]

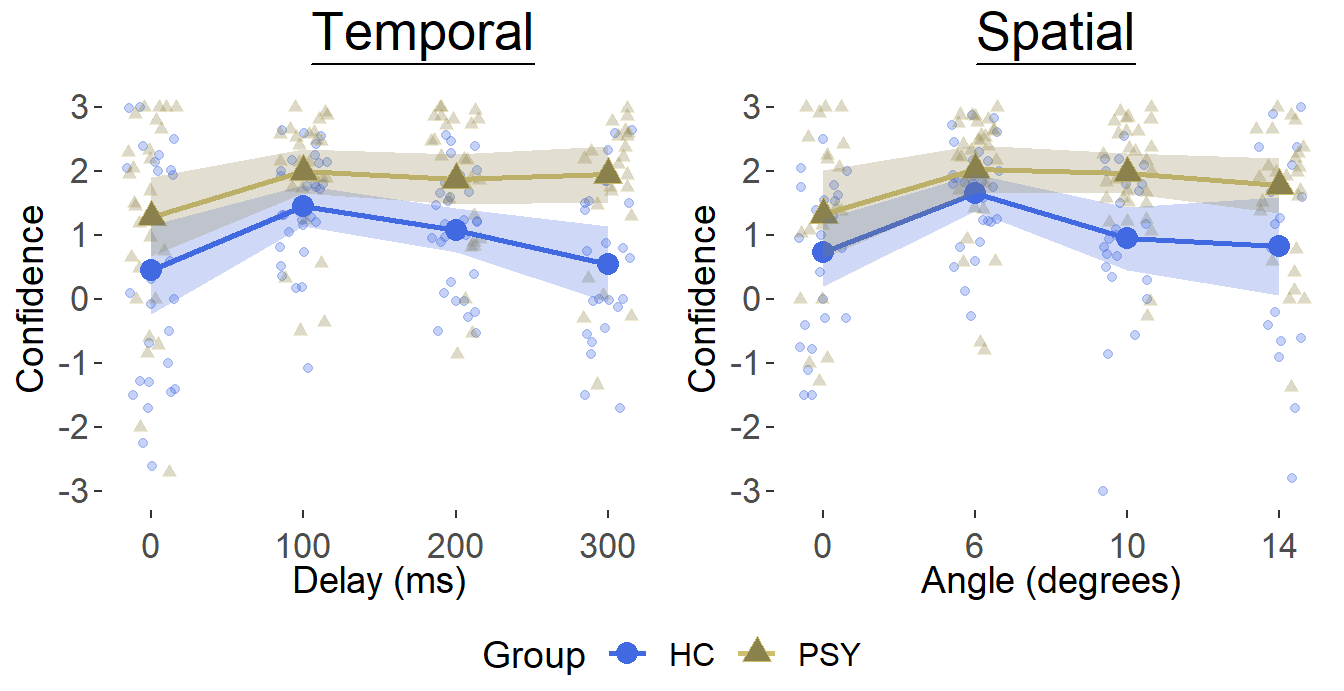

Supplement: Supplementary file 5 — Figure S3 [file 41537_2022_212_MOESM5_ESM.tif]

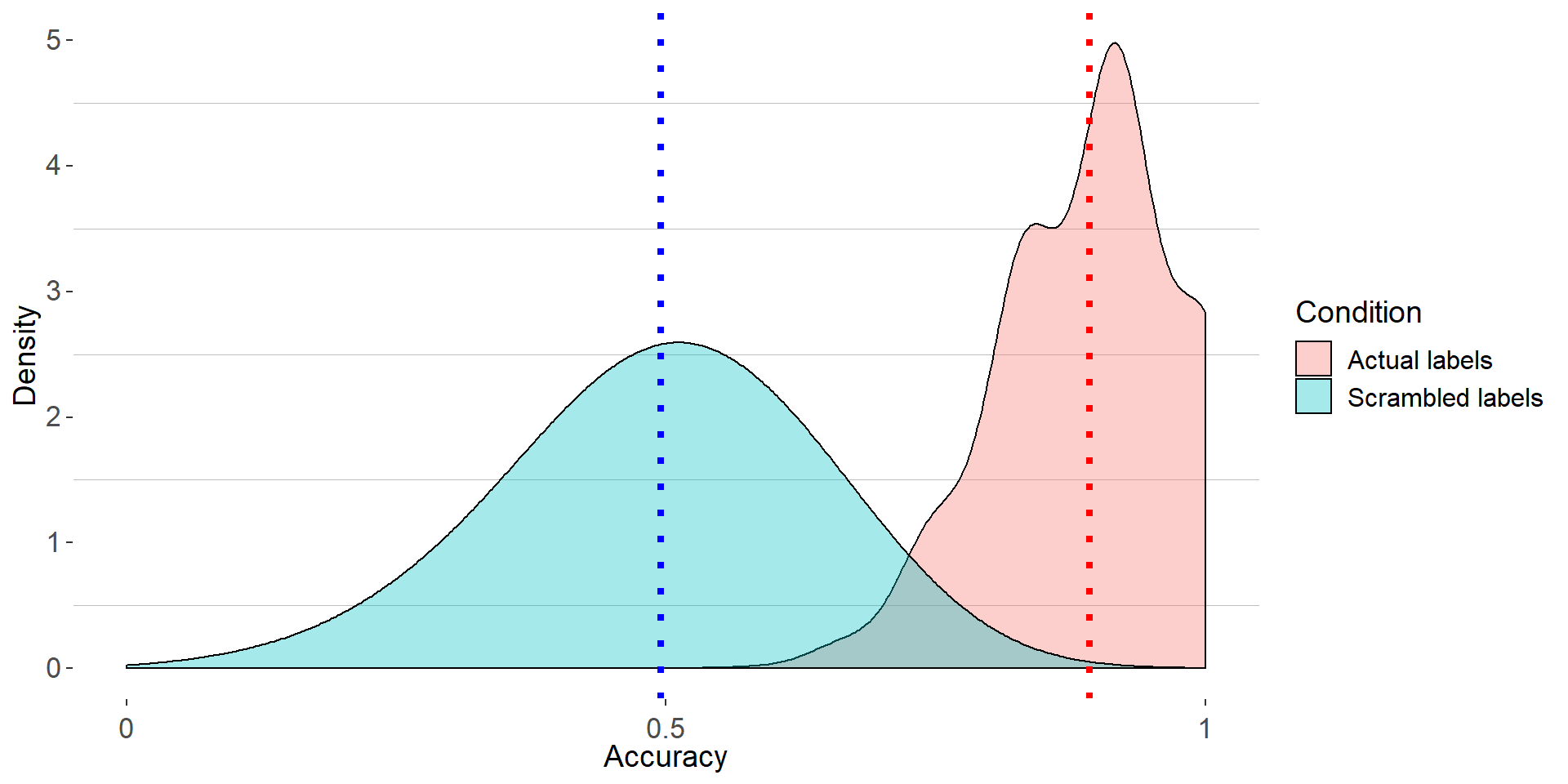

Supplement: Supplementary file 6 — Figure S4 [file 41537_2022_212_MOESM6_ESM.tif]
